# MFAFNet: A Lightweight and Efficient Network with Multi-Level Feature Adaptive Fusion for Real-Time Semantic Segmentation

**DOI:** 10.3390/s23146382

**Published:** 2023-07-13

**Authors:** Kai Lu, Jieren Cheng, Hua Li, Tianyu Ouyang

**Affiliations:** 1 School of Cyberspace Security (School of Cryptology), Hainan University, Haikou 570228, China; 2 Department of Public Safety Technology, Hainan Vocational College of Political Science and Law, Haikou 571100, China; 3 School of Computer Science and Technology, Hainan University, Haikou 570228, China; 4 Faculty of Engineering, The University of Sydney, Camperdown, NSW 2006, Australia

**Keywords:** semantic segmentation, real time, feature fusion, lightweight model

## Abstract

Currently, real-time semantic segmentation networks are intensely demanded in resource-constrained practical applications, such as mobile devices, drones and autonomous driving systems. However, most of the current popular approaches have difficulty in obtaining sufficiently large receptive fields, and they sacrifice low-level details to improve inference speed, leading to decreased segmentation accuracy. In this paper, a lightweight and efficient multi-level feature adaptive fusion network (MFAFNet) is proposed to address this problem. Specifically, we design a separable asymmetric reinforcement non-bottleneck module, which designs a parallel structure to extract short- and long-range contextual information and use optimized convolution to increase the inference speed. In addition, we propose a feature adaptive fusion module that effectively balances feature maps with multiple resolutions to reduce the loss of spatial detail information. We evaluate our model with state-of-the-art real-time semantic segmentation methods on the Cityscapes and Camvid datasets. Without any pre-training and post-processing, our MFAFNet has only 1.27 M parameters, while achieving accuracies of 75.9% and 69.9% mean IoU with speeds of 60.1 and 82.6 FPS on the Cityscapes and Camvid test sets, respectively. The experimental results demonstrate that the proposed method achieves an excellent trade-off between inference speed, segmentation accuracy and model size.

## 1. Introduction

Semantic segmentation is a basic but challenging task in computer vision that aims to predict the class label of each pixel point in an image. It plays a crucial role in the deep understanding of various objects in urban street scenes and is widely used in areas such as autonomous driving [1], robotics perception [2], and medical image analysis [3]. Robustness and accuracy are crucial for vision tasks [4]. Traditional image segmentation methods, such as conditional random field and k-mean clustering, have poor robustness and low accuracy, and it is difficult to extract semantic and spatial features of objects stably. Deep learning techniques can address this problem better. In recent years, since deep learning methods are rapidly developing and have achieved remarkable results in the field of engineering [5,6,7,8,9], semantic segmentation models based on deep learning have gradually become mainstream. For applications such as self-driving cars and mobile devices that have limited computational resources and require efficient interactions, strict inference speed and segmentation accuracy are usually required to achieve fast actions. While complex semantic segmentation models have excellent performance in terms of segmentation accuracy, they suffer from the problems of high computational power and slow inference speed, which cannot meet the needs of low-performance IoT devices for real-time tasks. Therefore, it is necessary to design an efficient and lightweight real-time semantic segmentation method to achieve a better speed–accuracy trade-off.

To fulfill the demand of real-time interaction, real-time semantic segmentation methods have emerged in recent years and achieved remarkable results. These methods can be broadly classified into two categories: (1) Module optimization first extracts features based on a lightweight real-time backbone network, and later builds an efficient context information aggregation module to improve segmentation accuracy. For example, FPANet [10] uses ResNet18 as the feature encoder, and then builds the feature pyramid fusion module (FPFM) in the decoding stage to fully fuse the output features at different levels. DFFNet [11] and MIFNet [12] are all based on the lightweight MobileNetV2 network as feature encoder, and in order to capture multi-scale contextual information to improve the segmentation accuracy of the network, propose the lightweight semantic pyramid module and lightweight information fusion module, respectively. (2) Architecture design uses optimized convolution to design bottleneck module to reduce the computational cost of the network and improve the segmentation accuracy. For example, ESNet [13], LRDNet [14], and LAANet [15] build network architectures using asymmetric convolution, dilated convolution, and depth-wise convolution to extract context information with fewer network parameters and computational cost. Although the above works build lightweight models that effectively reduce the computational overhead, for the former, the modules provide limited performance improvements and the number of network parameters is difficult to reduce further. For the latter, the optimized convolution reduces the feature extraction capability of the network, which leads to a significant reduction in segmentation accuracy. Therefore, achieving a balance between model size, inference speed, and segmentation accuracy is still an open research problem.

**Figure 1:**
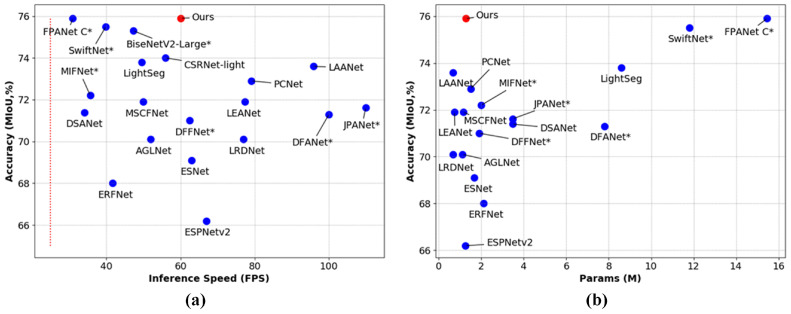
Performance comparison on the Cityscapes test set. (**a**) Speed–accuracy, (**b**) model size–accuracy. Red dot indicates our method, while blue dots mean other methods. The red line represents the real-time speed. “*” indicates that the corresponding method is pre-trained on other datasets. Our method achieves the most advanced trade-off between inference speed, segmentation accuracy, and model size.

To solve the problem of the balance between inference speed, model size and segmentation accuracy, we propose a lightweight and efficient network with multi-level feature adaptive fusion for real-time semantic segmentation, called MFAFNet. The MFAFNet is an asymmetric encoder–decoder structure. In the encoder, we design an efficient separable asymmetric reinforcement non-bottleneck (SAR-nbt) module, which first captures short- and long-range features through a parallel structure, and then fuses the multi-scale features and enhances the representations. The parallel structure takes advantage of asymmetric convolution, depth-wise convolution and dilated convolution to reduce the number of parameters and computational overhead of the network. In the decoder, we propose a feature adaptive fusion module (FAFM) that fuses low-level, mid-level, and high-level features to reduce the loss of spatial detail information caused by multiple down-sampling. To reduce the semantic gaps existing between multi-level features, the channel attention and spatial attention representation maps of the mid-level features are constructed, which are applied to the low-level and high-level features to draw closer the relationship between the three branches. Extensive quantitative and qualitative evaluations demonstrate that the proposed approach achieves competitive results on the Cityscapes test set compared to state-of-the-art real-time semantic segmentation methods as shown in Figure 1.

The main contributions of this paper are summarized as follows:

- We propose a separable asymmetric reinforcement non-bottleneck module. This module makes full use of the optimized convolution to reduce the number of parameters and increase the inference speed, and constructs a parallel structure to extract short- and long-range information to obtain multi-scale features. Then, the channel attention weights of the multi-scale feature maps are computed for feature re-calibration.
- We propose a feature adaptive fusion module that effectively balances the multi-level feature maps to reduce the loss of spatial detail information. It also combines spatial attention and channel attention that adaptively enhances multi-level features to reduce the semantic gaps existing in multiple resolution feature maps.
- We design a real-time semantic segmentation network to achieve competitive segmentation accuracy with lower computational overhead and fewer network parameters, achieving a trade-off between inference speed, segmentation accuracy and model size.

The remainder of this paper is organized as follows. Section 2 reviews the related work. Section 3 presents the design details of our proposed MFAFNet and related components. Section 4 conducts the experiments and analyzes the results. Section 5 concludes the work.

## 2. Related Work

### 2.1. Real-Time Semantic Segmentation

Real-time semantic segmentation aims to achieve high-quality segmentation results with low computational overhead and is often used in IoT devices that lack computational resources. In recent years, there have been many deep learning works devoted to real-time semantic segmentation research. ESNet [13], ERFNet [16], and LEANet [17] constructed encoding and decoding architectures with asymmetric convolution and dilated convolution to further reduce the number of network parameters to maintain a balance of speed and accuracy. Fast-SCNN [18] and BiSeNetV2 [19] proposed a two-branch or multi-branch network that captures semantic information using deep semantic branches, reduces the loss of spatial details using information branches, and then fully integrates high-level semantic and spatial detail information to improve the segmentation accuracy with lower computational overhead. STDC [20] modified and optimized the backbone network of BiSeNet by designing a more efficient short-term dense connectivity module, which reduces the computational effort in the network while extracting rich feature information. Although these methods can achieve high accuracy and real-time inference speed, there is still room for further improvement on the real-time semantic segmentation task.

### 2.2. Multi-Scale Feature Fusion

Different layers in the segmentation model contain different semantic information. The shallow network contains rich spatial detail information, and the deep network has a strong ability to characterize semantic information. Fusing the features of deep and shallow layers can effectively improve the accuracy of the network [21]. In addition, global semantic information can provide clues to segment the category distribution, and fusing global and local features can improve the robustness of the model.

The current multi-scale feature fusion methods mainly include two types: parallel multi-branching structures and jump-connected structures. The former is mainly used to fuse local and global features, for example, PSPNet [22] proposed a pyramid pooling module (PPM), which extracted multi-scale features by pooling operations at different scales to reduce the loss of contextual information characterizing the relationships between different sub-regions. DeepLabv3 [23] designed an atrous spatial pyramid pooling (ASPP) module that captured multi-scale contexts with different dilated convolution expansion rates to further integrate multi-scale features. MIFNet [12] and DFFNet [11] are optimized for the ASPP module, utilizing operations such as dense join, depth-wise convolution, and dilated convolution to efficiently integrate multi-scale information. The latter is mainly used to fuse shallow and deep features, e.g., LAANet [15] proposed the attention-guided feature fusion upsampling (AFFU) module, which combined the spatial attention map generated by shallow features and the channel attention map generated by deep features to efficiently fuse the output features of different layers in the encoding stage. EFRNet [24], FPANet [10] and JPANet [25] reduced the loss of deep feature spatial detail information by constructing efficient feature fusion modules to aggregate outputs of different depths in the network with strong semantic information.

## 3. Proposed Method

In this section, we first overview the architecture of the proposed network. Then, the design details of the main components of the network, the separate asymmetric reinforcement non-bottleneck (SAR-nbt) module and the feature adaptive fusion module (FAFM) are presented.

### 3.1. Network Overview

**Figure 2:**
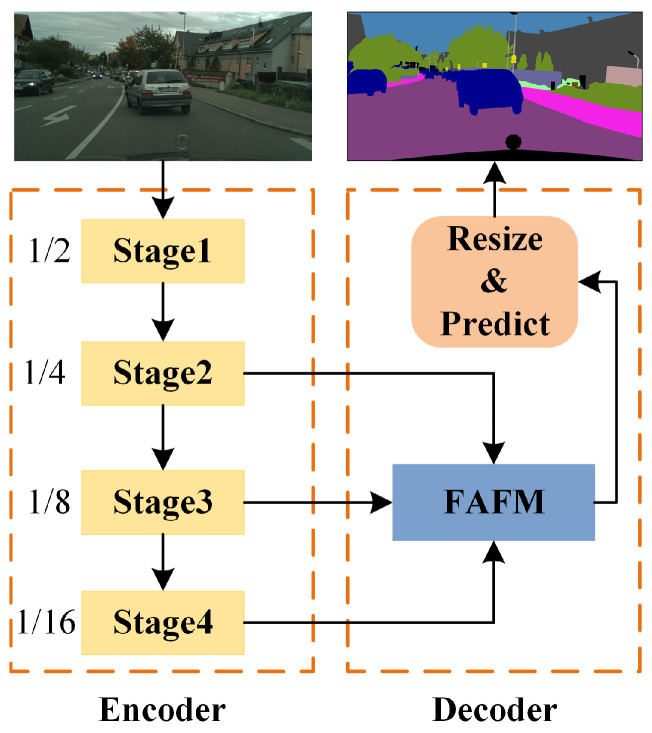
Overview architecture of the proposed MFAFNet.

The overall network architecture of MFAFNet is shown in Figure 2, which is an asymmetric encoder–decoder structure. The encoder contains four down-sampling operations, decreasing the resolution to 1/16 to reduce the computational overhead of the feature extraction process. The decoder contains a feature adaptive fusion module (FAFM), which fuses the output features of multi-levels in the encoding stage to reduce the loss of spatial detail information and improve the segmentation accuracy.

**Figure 3:**
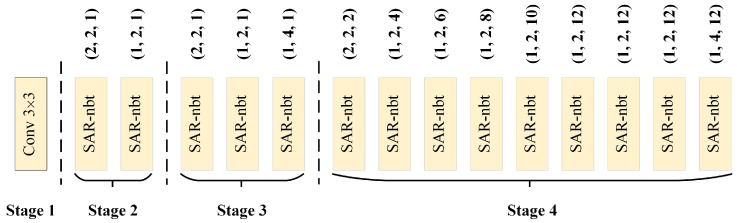
The structure of the encoder. The three digits besides the SAR-nbt indicate the stride, the channel expansion factor, and the dilatation rate of DDW, respectively. The detailed information of the SAR-nbt is shown in Figure 4.

The design details of the encoder are shown in Figure 3, which consists of four stages. Stage 1 uses a 3×3 standard convolution with stride of 2 to achieve the first down-sampling, which quickly reduces the resolution of the input features and reduces the computational complexity. The 2nd, 3rd and 4th stages are composed of SAR-nbt modules, containing 2, 3, 9 SAR-nbt modules, respectively, and down-sampling only happens in the first SAR-nbt module of each stage. To avoid weakening the feature-extraction capability of the network due to the use of depth-wise convolution, the channel expansion is added to the SAR-nbt module, and the expansion factor is 4 only for the last one in stage 3 and stage 4, and 2 for the others so as to avoid a too-wide channel dimension that would substantially increase the computational cost. In addition, in order to expand the receptive field to extract context information at a distance and to avoid losing semantic information by introducing dilated convolution prematurely, dilated convolution is introduced in the middle layer of the network. The SAR-nbt module in stage 4 introduces dilated convolution to gradually expand the receptive field, and the dilation rates are set to 2, 4, 6, 8, 10, 12, 12, 12, and 12, respectively.

For faster reasoning speed, many networks directly use bilinear interpolation methods to recover deep semantic features to the original image resolution, and although this operation improves efficiency, it leads to the loss of spatial detail information and loss of accuracy. While the decoder structure of the progressive layer-by-layer fusion of shallow features performs better in terms of segmentation accuracy, the negative effects, such as slower inference and increased computational cost, are also evident. Therefore, we design a feature adaptive fusion module in the decoder, as shown in Figure 5, to efficiently fuse multi-level features to compensate for the loss of spatial information caused by the high abstraction of deep features and multiple down-sampling, and to significantly improve the segmentation accuracy with only a small computational overhead. Finally, we use 1×1 convolution and bilinear interpolation to recover the features to the original input size to predict the final segmentation result.

### 3.2. Separable Asymmetric Reinforcement Non-Bottleneck Module

In recent years, many lightweight real-time semantic segmentation networks [14] have adopted the residual structure when designing bottleneck blocks and used optimized convolution to replace the standard convolution of the original residual blocks to reduce the number of network parameters and computational overhead.

**Figure 4:**
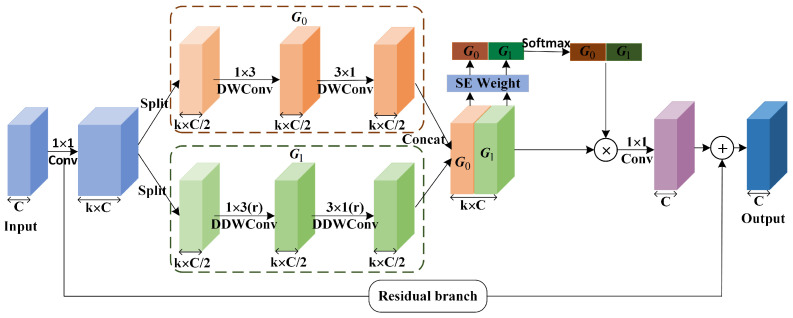
The structure of the proposed separable asymmetric reinforcement non-bottleneck (SAR-nbt) module. “DWConv” indicates depth-wise convolution. “DDWConv” indicates dilated depth-wise convolution.

Optimized convolution includes two categories: factorized convolution and dilated convolution. Among them, factorized convolution is mainly used to reduce the number of parameters and the computational overhead, such as group convolution [26], asymmetric convolution [16], and depth-wise separable convolution [27]. Dilated convolution [23] can effectively expand the receptive field of the convolution kernel to capture more contextual information without increasing the number of parameters. In addition, there are many works that combine multiple optimized convolutions to further improve the performance of convolution, such as dilated depth-wise separable convolution [11], asymmetric depth-wise separable convolution [14], and asymmetric dilated depth-wise separable convolution [15]. Assuming that the size of the input feature map is $H\times W\times C_{in}$, the size of the convolution kernel is k×k, the size of the output feature map is $H\times W\times C_{out}$, and the dilation rate is *d*, regardless of bias. The comparison of the parameters and receptive fields between several common optimized convolutions and standard convolutions is shown in Table 1. Obviously, the optimized convolution can effectively reduce the number of parameters of the residual blocks, and obtain a larger receptive field. Inspired by the success of aforementioned works, we take advantage of asymmetric dilated depth-wise separable convolution to design a separable asymmetric reinforcement non-bottleneck (SAR-nbt) module as shown in Figure 4.

The SAR-nbt module is mainly implemented in six steps. Each convolutional layer in the SAR-nbt module is followed by one batch normalization layer and one PReLU activation layer (the PReLU activation function has been demonstrated in many works to perform excellent in lightweight networks). First, to avoid the problem of feature encoding capability degradation due to the use of depth-wise convolution in the network, the SAR-nbt module first uses 1×1 convolution to expand the number of channels, expanding the channels to k times the original channels to improve the feature extraction capability. Second, in order to reduce the computational complexity of the network, a channel-splitting operation is used to divide the boosted feature maps into two equal groups along the channels: $G_{0}$ and $G_{1}$. Third, multi-scale features are extracted by two parallel groups. Many works demonstrated that fusing multi-scale features can effectively improve segmentation accuracy, so different scale features are extracted from two groups separately. $G_{0}$ uses an asymmetric depth-wise convolution to capture short-range context information. Specifically, a standard 3×3 depth-wise convolution is decomposed into a 1×3 convolution and a 3×1 convolution, an operation that reduces the number of network parameters without reducing the receptive field. $G_{1}$ further incorporates dilated convolution to effectively expand the receptive field and extract long-range context information without increasing the computational overhead. Specially, when the stride of the SAR-nbt module is set to 2, it indicates that the stride of the 1×3 convolution in $G_{0}$ and $G_{1}$ is 2, which achieves down-sampling of the input feature maps. After that, the features of the two groups are fused using the channel concatenate operation to obtain rich multi-scale features and adapt to the recognition of objects of different sizes.

Fourth, the multi-scale feature maps are enhanced by using channel attention SENet. Specifically, the channel-wise attention vector is obtained by using the SEWeight module to extract the attention of the feature maps with different scales, and the attention vector is re-calibrated by using the Softmax. Then, the re-calibrated weights are multiplied with the corresponding feature maps to achieve the enhancement of key features and obtain the refined feature maps. Fifth, the number of channels of the refined feature maps is compressed using a 1×1 point-wise convolution to keep the same number of channels as the input feature maps. Finally, a residual branch is constructed to fuse the initial input with the output to solve the problem of gradient disappearance and explosion during the training process. Specifically, when the stride of the SAR-nbt module is 1, the residual branch represents the input feature map. When the stride = 2, the residual branch contains an average pooling layer, a 1×1 convolutional layer, and a batch normalization layer to act on the input to keep the resolution of the residual branch consistent with the output feature map.

### 3.3. Feature Adaptive Fusion Module

High-level features contain rich semantic information and have high abstract generalization; low-level features have rich spatial detail information and can guide object position features. Making full use of the deep semantic information and the shallow spatial detail information can effectively improve the segmentation accuracy. Most existing real-time semantic segmentation methods use element addition or channel concatenation operations when performing feature fusion, ignoring the existence of semantic gaps in features at different levels. To effectively fuse information from multi-levels, we propose a feature adaptive fusion module (FAFM), which adaptively enhances features at different levels through an attention mechanism to reduce the semantic gap existing between low-level and high-level features.

**Figure 5:**
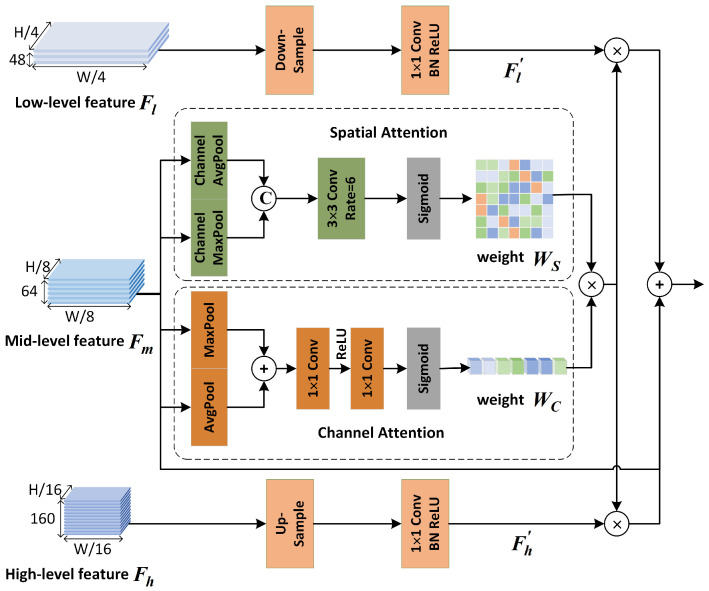
Design details of feature adaptive fusion module.

The FAFM module is shown in Figure 5. *H* and *W* represent the height and width of the input RGB image. The output features of stage 2, stage 3, and stage 4 are used in the fusion process, corresponding to the low-level feature $F_{l}$, the mid-level feature $F_{m}$, and the high-level feature $F_{h}$, and the feature map sizes are (W/4)×(H/4)×48, (W/8)×(H/8)×64, and (W/16)×(H/16)×160, respectively. To avoid excessive computational overhead, an element addition operation is used to fuse multi-level features. And the number of channels and resolution of each level feature maps are different; therefore, the consistency processing of each level feature is needed before feature fusion. Considering that the mid-level features have better semantic information and also retain spatial information, the number of channels and resolution of the middle-level features are chosen as the benchmark. The low-level features are down-sampled using the averaging pooling operation, and the number of channels is expanded using 1×1 point-wise convolution, while the high-level features are up-sampled using the bilinear interpolation method. The number of channels is compressed using 1×1 point-wise convolution, and the processed low-level feature $F_{l}^{{}^{\prime }}$ and high-level feature $F_{h}^{{}^{\prime }}$ are obtained in turn, both with feature map size (W/8)×(H/8)×64. The above operations can be expressed as follows:

(1) $$F_{l}^{{}^{\prime }}=f^{1\times 1}\left(Down\left(F_{l}\right)\right)$$

(2) $$F_{h}^{{}^{\prime }}=f^{1\times 1}\left(Up\left(F_{h}\right)\right)$$

where Down and Up indicate the down-sampling and up-sampling operations, respectively. $f^{k\times k}$ refers to the standard convolution with a kernel size of *k*, with the same meaning for subsequent references.

Then, to reduce the semantic gaps existing in different level features, the channel attention weights $W_{c}$ and spatial attention weights $W_{s}$ of mid-level features are obtained to enhance the representations of other level features. The attention weights $W_{c}$ and $W_{s}$ are calculated as follows:

(3) $$W_{c}=\sigma \left(f^{1\times 1}\left(R\left(f^{1\times 1}\left(P_{max}^{s}\left(F_{m}\right)+P_{avg}^{s}\left(F_{m}\right)\right)\right)\right)\right)$$

(4) $$W_{s}=\sigma \left(f^{3\times 3}\left(C\left[P_{max}^{c}\left(F_{m}\right),P_{avg}^{c}\left(F_{m}\right)\right]\right)\right)$$

where $P_{max}^{s}$ and $P_{avg}^{s}$, and $P_{max}^{c}$ and $P_{avg}^{c}$ represent the two sets of max-pooling and average-pooling operations along the spatial and channel axes, respectively. C[.,.] represents the channel concatenation operation. *R* and σ represent the ReLU activation function and the sigmoid activation function, respectively. The ReLU and sigmoid activation functions are simple to implement and easy to optimize, and are still used by most attention mechanisms to compute attention weights.

Then, both channel attention weights $W_{c}$ and spatial attention weights $W_{s}$ are multiplied with low-level features and high-level features to achieve the adaptive adjustment of features at different levels. Finally, the multi-level features are element-wise added and fused, and the 3 × 3 convolutional layer is used to make the feature fusion more fully, and the final output of the FAFM module is obtained. The final features $F_{out}$ can be obtained according to the following formula:

(5) $$F_{out}=f^{3\times 3}\left(F_{l}^{{}^{\prime }}\times W_{c}\times W_{s}+F_{m}+F_{h}^{{}^{\prime }}\times W_{c}\times W_{s}\right)$$

Obviously, the proposed FAFM module uses an attention mechanism to reduce the semantic gaps that exist between multi-levels, effectively balances feature maps with multiple resolutions, and improves segmentation accuracy.

## 4. Results

### 4.1. Datasets and Evaluation Metrics

#### 4.1.1. Datasets

**Cityscapes dataset [28].** The Cityscapes dataset is a large-scale image dataset on the semantic understanding of urban street scenes. It is mainly derived from a set of video sequences recorded in 50 different urban driving scenes and consists of 5000 high-quality finely annotated images and 20,000 coarsely annotated images. The dataset contains semantic, instance and dense pixel annotations for 30 categories, 19 of which are used for training and evaluation. We use only 5000 finely annotated images for experiments, of which 2975 are used for training, 1525 for testing, and 500 for validation.

**Camvid dataset [29].** Camvid is another dataset for road and driving scene understanding. It contains a total of 701 images, which can be divided into three groups, the training set, validation set, and test set, each containing 367, 101, and 233 images. The dataset contains 11 different classes, and the resolution is 960 × 720.

#### 4.1.2. Evaluation Metrics

**Accuracy.** The accuracy of semantic segmentation is mainly measured by the mean intersection over union (MIoU), where MIoU refers to the mean of the ratio between the intersection and the union of the predicted segmentation maps and the true labels for all categories. Assuming that *k* is the number of segmentation categories, MIoU can be defined as follows:

(6) $$MIoU=\frac{1}{k}\sum_{i=0}^{k}\frac{P_{ii}}{\sum_{j=0}^{k}P_{ij}+\sum_{j=0}^{k}P_{ji}-P_{ii}}$$

where $P_{ij}$ denotes the number of pixels inferred from category *i* to category *j*, and $P_{ii}$ denotes the true examples. $P_{ij}$ and $P_{ji}$ refer to false-positive and false-negative examples, respectively.

**Inference speed.** FPS is an image domain definition of the number of frames per second of the transmitted images, which is often used to indicate the real-time performance of a model.

**Network parameters.** The total number of weights and biases for each layer in the network.

**Computational complexity.** It is used to measure the number of floating-point operations (FLOPs) in the model to evaluate the speed and efficiency of the model.

### 4.2. Implementation Details

We used Pytorch 1.9 and NVIDIA 3090 GPU for all experiments. We followed the strategies proposed by RegSeg [30]. We utilized the “poly” learning rate policy, in which the initial rate is multiplied by ${\left(1-\frac{iter}{max_{iter}}\right)}^{power}$. The power was set to 0.9, the initial learning rate was set as 0.05, and the weight decay was 0.0005. In addition, a linear warm-up with a learning rate of 0.1 to 1 time was used for the first 3000 iterations during training. The data-augmentation method included random horizontal flip, random scaling to [400, 1600] and random cropping to [768, 768]. It also used a set of reduced random augmentation operations (auto contrast, equalization, rotation, color, contrast, brightness, and sharpness), and two random operations of the 0.2 level were applied to each image. To accelerate the training speed without losing accuracy, mixed precision training was used. The loss function used in the training process is the online hard example mining loss (OHEM) [31], the batch size is 8, and a total of 1000 epochs were trained. When submitting to the test server, we trained on the trainval set.

When training on the Camvid dataset, the training strategy was similar to that on the Cityscapes dataset. Since the model is not pre-trained on other datasets, and the Camvid dataset is too small, it may not be possible to fit the network. Therefore, the training results on the Cityscapes dataset were used as pre-training weights for the Camvid dataset. The data were augmented using random level flipping, random scaling to [288, 1152], with a batch size of 12. A total of 200 epochs were trained without using the random augmentation strategy.

### 4.3. Ablation Study

In this section, we design a series of experiments to demonstrate the effectiveness of our method. All ablation experiments were conducted on the Cityscapes dataset.

#### 4.3.1. Ablation for SAR-nbt Module

To study the effect of the attention mechanism on the feature extraction ability of the network, we first conducted the SE attention ablation study on the backbone network, and the results are shown in Table 2. The SAR-nbt module that used the SE attention mechanism improved the accuracy by 1.51% with only 0.28 M additional parameters, and achieved a segmentation accuracy of 77.08% on the Cityscapes validation set, which indicated that the simple attention mechanism can significantly improve the ability to extract contextual information with a small amount of computational cost.

In addition, to explore the effect of dilated convolution and to select the appropriate dilation rates to better capture long-range semantic information and improve model performance, we designed dilation rate ablation experiments for the SAR-nbt module. We only used the dilated convolution in the SAR-nbt module of stage 4, and the left branch captured the short-range features by default, so only the convolution block of the right branch was filled with the dilation rate. Table 2 shows the comparison results under different dilation rates. It can be seen that the dilated convolution did not increase the number of network parameters, and the segmentation accuracy was highest for the settings with the dilation rate list of 2, 4, 6, 8, 10, 12, 12, 12, and 12. The segmentation accuracy was the lowest for the settings with dilation rates of 2, 6, 6, 12, 12, 18, 18, 24, 24. Due to the large dilation rates, too much noise information may be introduced, which leads to the poor information capture ability of the convolutional kernel and reduces the segmentation accuracy. Finally, the proposed MFAFNet used the highest segmentation accuracy with the dilation rate list setting, and the ablation of subsequent modules was performed based on this setting.

#### 4.3.2. Ablation for FAFM Module

To verify the efficiency of the proposed FAFM module, ablation experiments with different branch fusion strategies were performed. In order to reduce the training time, the ablation experiments of the FAFM module trained 500 epochs. Table 3 shows the comparison results of different fusion methods, where x16, x8, and x4 indicate the down-sampling multiplier of the feature map of the branch compared to the original input image size, corresponding to the output of stage 4, stage 3, and stage 2, respectively. As shown in Table 3, the strategy with branch fusion showed a significant improvement in segmentation accuracy compared to predicting x16 (without the fusion module) directly, which proves that the shallow features can effectively compensate for the loss of spatial detail information of the deep features. And the FAFM module had the highest accuracy, compared with the direct prediction of x16 branch features; only 0.05 M number of parameters and 0.36 G floating point operations were increased, and the MIoU was improved by 4.41%.

**Figure 6:**
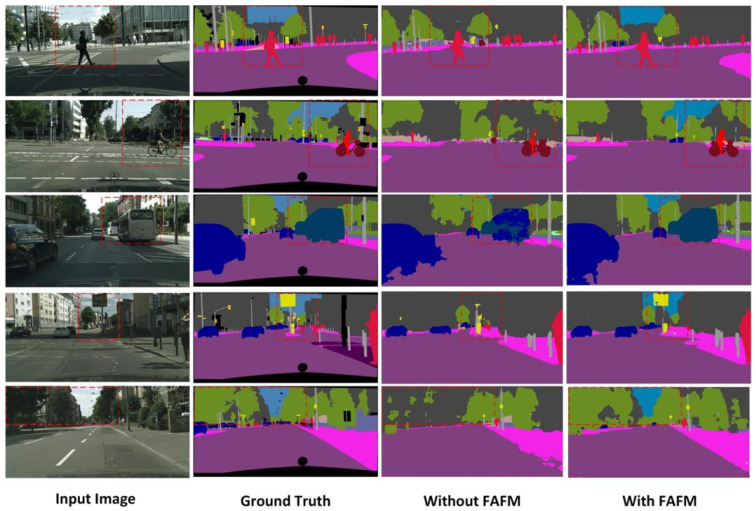
Visualization results of the FAFM module on Cityscapes validation set.

Figure 6 displays the visualization results of the FAFM module on the Cityscapes validation set, with the red dashed boxes marking the parts of the same image where the segmentation accuracy contrast is more obvious. As can be seen, for the results without the FAFM module, such as the sky in the first image, the car in the third image, and the traffic sign in the fourth image, after a series of encoding and decoding operations, the result prediction directly by the high-level feature maps lost the original spatial detail information of the object. The method that uses the FAFM module may also suffer from the problem of inconspicuous object edge segmentation, but it can basically retain the original spatial information of the objects and achieve better segmentation results. In summary, the proposed FAFM module can efficiently fuse the multi-level output feature maps in the encoder to reduce false predictions and substantially improve the accuracy of the object segmentation with less computational overhead.

**Figure 7:**
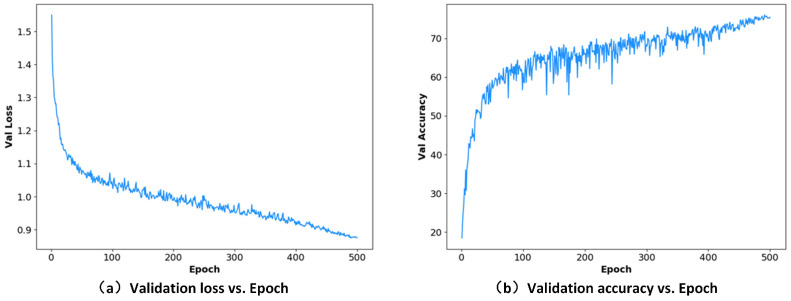
The validation loss curve and accuracy curve of MFAFNet during 500 training epochs on Cityscapes validation set.

Finally, we conducted comparative ablation experiments of the modules to verify the effectiveness of each module in the model structure. We considered the backbone without using the dilated convolution (dilation rate of 1 in all SAR-nbt modules) and without the FAFM module as the baseline. As shown in Table 4, compared with the baseline method, the SAR-nbt (with dilated convolution ) and FAFM module increased only 0.05 M parameters and 0.36 GFLOPs, which was able to achieve a 9.78% improvement in MIoU. In addition, to clearly illustrate the training process, Figure 7 intuitively shows the validation loss curve and accuracy curve of MFAFNet on the Cityscapes validation set. The loss curve dropped smoothly and converged eventually, indicating that our MFAFNet can be well trained. In general, qualitative and quantitative analyses proved the effectiveness of SAR-nbt and the FAFM module. The proposed MFAFNet can achieve better efficiency and an accurate balance.

### 4.4. Comparison with Other State-of-the-Art Methods

#### 4.4.1. Results on Cityscapes

This section reports some performance results of MFAFNet on the Cityscapes dataset, such as model size, accuracy, inference speed, etc. It is then compared and analyzed with other state-of-the-art real-time semantic segmentation methods to illustrate the efficiency of MFAFNet. In this section, GTX 1080Ti GPU is used uniformly for testing the inference speed.

Table 5 shows the comparison results; our proposed MFAFNet was not pre-trained with any other dataset and contained only 1.27 M number of parameters, achieving 75.9% MIoU on the Cityscapes test set. Moreover, for input images with a resolution of 512×1024, MFAFNet achieved an inference speed of 60.1 FPS on a single 1080Ti GPU. Compared with the excellent real-time semantic segmentation methods in recent years, our MFAFNet achieved a more superior performance. Specifically, compared with MIFNet [12], DSANet [32], LightSeg [33], and CSRNet-light [34], the proposed MFAFNet not only has a more lightweight network structure and faster inference speed but also has a higher segmentation accuracy. Our method was also 5.8% and 2.3% higher than LRDNet [14] and LAANet [15] in MIOU. Compared with SwiftNet [35] and FPANet [10], which also have high accuracy, our MFAFNet has the highest accuracy with at least 9 times fewer parameters and is not pre-trained on other large datasets. In conclusion, compared with other segmentation methods, our proposed MFAFNet has superior performance, and achieved real-time inference speed while significantly improving the segmentation accuracy with a smaller number of parameters, and achieved a better trade-off between model size, inference speed and segmentation accuracy.

**Figure 8:**
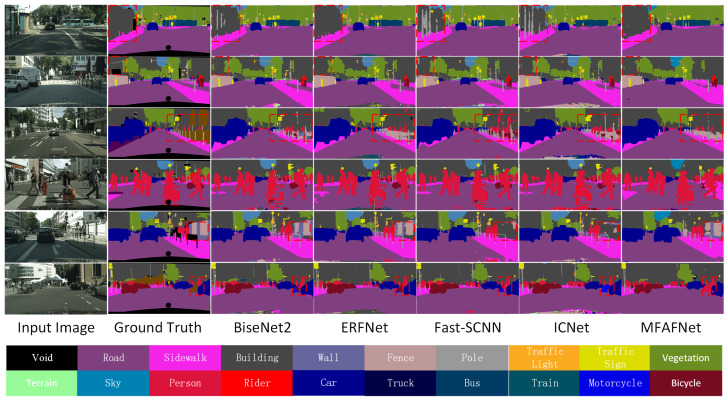
Some visual comparisons on the Cityscapes validation set. From left to right are input image, ground truth, predicted results from BiseNet2, ERFNet, Fast-SCNN, ICNet, and our MFAFNet.

We visually compared the prediction results of different methods on the Cityscapes validation set as shown in Figure 8. To facilitate comparison, we use red boxes in Figure 8 to mark areas where segmentation errors are more obvious among all methods. It can be seen that our algorithm is very competitive among the current excellent algorithms.

In Table 6, we further evaluated the IoU (%) performance for each category on the Cityscapes test set. Our method achieved better segmentation results on most of the categories, with the highest IoU scores in 16 out of 19 categories, such as roads, buildings, cars, skies, and traffic lights. Our method has better recognition ability for objects of different sizes with an average accuracy of 75.9% for the categories, which fully illustrates the efficiency of the MFAFNet.

#### 4.4.2. Results on Camvid

In this subsection, to verify the robustness and effectiveness of the model, we evaluate the performance of our method on the Camvid test set. And then, it is compared with several state-of-the-art real-time semantic segmentation models. The comparison results are shown in Table 7, and it can be seen that our method can still achieve better segmentation results on the Camvid dataset. Our method is 4.4% higher than CGNet [41] in MIOU. In addition, the proposed method has the highest accuracy and is equally lightweight compared to recent real-time segmentation methods, such as MIFNet [12], JPANet [25], and LEANet [17]. Therefore, our method still maintains good accuracy (69.9% MIoU), a lightweight model size (less than 1.3 M) and fast inference speed (82.6 FPS on a 640×360 resolution image) compared to most existing semantic segmentation methods.

## 5. Conclusions

In this paper, a lightweight and efficient multi-level feature adaptive fusion network for real-time semantic segmentation is proposed, named MFAFNet. The MFAFNet is an asymmetric encoding and decoding structure, and in the encoder, we design a separable asymmetric enhancement non-bottleneck (SAR-nbt) module that makes full use of the optimized convolution to increase the inference speed, and constructs a parallel structure to extract short- and long-range information to obtain multi-scale features. In the decoder, we propose a feature adaptive fusion module that effectively balances the multi-level feature maps to reduce the loss of spatial detail information. It also combines spatial attention and channel attention to adaptively enhance multi-level feature maps and reduce the semantic gaps that exist at multi-levels during feature fusion. To verify the effectiveness of the proposed MFAFNet, we evaluate the performance on two challenging datasets with state-of-the-art real-time semantic segmentation methods. Experimental results demonstrate that our method achieves a trade-off between inference speed, segmentation accuracy, and model size.

Although the proposed MFAFNet achieves excellent segmentation accuracy in the field of real-time semantic segmentation and can basically restore the spatial location of the object, there is still room for further improvement in the segmentation of the object edges. Our method only extracts the spatial domain features of the image but ignores the effect of the frequency domain information. The high-frequency component of the frequency domain information contains rich edge outline information, which can be used reasonably to effectively improve the segmentation accuracy. In the future, our research will focus on how to combine frequency domain learning techniques in the network to further improve the accuracy of object edge segmentation.

## Figures and Tables

**Table 1:** Comparison of parameters and receptive field between common factorized convolutions and standard convolution.

Convolution Type	Parameters	Receptive Field
Standard convolution	$k^{2}C_{in}C_{out}$	k×k
Group convolution	$k^{2}C_{in}C_{out}/g$	k×k
Asymmetric convolution	$2kC_{in}C_{out}$	k×k
Depth-wise separable convolution	$k^{2}C_{in}+C_{in}C_{out}$	k×k
Dilated convolution	$k^{2}C_{in}C_{out}$	${\left(\left(k-1\right)d+1\right)}^{2}$
Dilated depth-wise separable convolution	$k^{2}C_{in}+C_{in}C_{out}$	${\left(\left(k-1\right)d+1\right)}^{2}$
Asymmetric depth-wise separable convolution	$2kC_{in}+C_{in}C_{out}$	k×k
Asymmetric dilated depth-wise separable convolution	$2kC_{in}+C_{in}C_{out}$	${\left(\left(k-1\right)d+1\right)}^{2}$

“*k*” denotes the size of the convolution kernel. “$C_{in}$” and “$C_{out}$” indicate the number of input and output channels, respectively. “*g*” represents the number of groups.

**Table 2:** Ablation study of SAR-nbt module on Cityscapes validation set.

Ablation Study	Type	Params	MIoU (%)
Attention	-	0.99	75.57
	SE Weight	1.27	77.08
Dilation rates	2, 4, 6, 8, 10, 12, 12, 12, 12	1.27	77.08
	2, 6, 6, 6, 6, 12, 12, 12, 12	1.27	76.67
	2, 4, 6, 8, 10, 12, 14, 16, 18	1.27	76.82
	2, 4, 4, 4, 4, 14, 14, 14, 14	1.27	76.36
	2, 6, 6, 12, 12, 18, 18, 24, 24	1.27	75.16

**Table 3:** Ablation study of FAFM module on the Cityscapes validation set.

Type	Params (M)	FLOPs (G)	MIoU (%)
x16	1.22	3.77	71.62
x16 + x8	1.26	4.10	74.95
x16 + x4	1.25	3.98	75.28
FAFM	1.27	4.13	76.03

**Table 4:** Ablation study of SAR-nbt and FAFM components on the Cityscapes validation dataset.

Baseline	SAR-nbt	FAFM	Params (M)	FLOPs (G)	MIoU (%)
✓			1.22	3.77	66.25
✓	✓		1.22	3.77	71.62
✓		✓	1.27	4.13	71.43
✓	✓	✓	1.27	4.13	76.03

**Table 5:** Performance comparison with state-of-the-art methods on Cityscapes test set.

Network	Resolution	GPU	Params (M)	FPS	MIoU (%)
ESPNet V2 [36]	512×1024	TitanX	1.25	67.0	66.2
ERFNet [16]	512×1024	TitanX	2.1	41.7	68.0
ESNet [13]	512×1024	1080Ti	1.66	63.0	69.1
ICNet ${}^{†}$ [37]	1024×2048	TitanX	26.5	30.3	69.5
AGLNet [38]	512×1024	1080Ti	1.12	52.0	70.1
LRDNet [14]	512×1024	1080Ti	0.66	77.0	70.1
DFFNet ${}^{†}$ [11]	512×1024	1080Ti	1.9	62.5	71.0
DSANet [32]	512×1024	1080Ti	3.47	34.08	71.4
JPANet ${}^{†}$ [25]	512×1024	1080Ti	3.49	109.9	71.6
LEANet [17]	512×1024	1080Ti	0.74	77.3	71.9
MSCFNet [39]	512×1024	TitanX	1.15	50	71.9
MIFNet ${}^{†}$ [12]	512×1024	1080Ti	2.0	35.7	72.2
PCNet [40]	1024×2048	2080Ti	1.5	79.1	72.9
LAANet [15]	512×1024	1080Ti	0.67	95.8	73.6
LightSeg [33]	512×1024	2080Ti	8.6	49.5	73.8
CSRNet-light [34]	512×1024	1080Ti	-	56.0	74.0
BiSeNetV2-L ${}^{†}$ [19]	512×1024	1080Ti	-	47.3	75.3
SwiftNet ${}^{†}$ [35]	1024×2048	1080Ti	11.8	41.0	75.5
EFRNet ${}^{†}$ [24]	512×1024	2080Ti	2.43	118.0	75.6
FPANet C ${}^{†}$ [10]	1024×1024	2080Ti	15.45	31.0	75.9
Ours	512×1024	1080Ti	1.27	60.1	75.9

“-” denotes that the model does not provide the corresponding result. “^†^” indicates that the corresponding method is pre-trained on ImageNet or Scratch dataset. The computational efficiency of GPU is TitanX < 1080Ti < 2080Ti.

**Table 6:** Per-class IoU (%) performance on the Cityscapes test set.

Method	MIoU	Road	Sidewalk	Building	Wall	Fence	Pole	Traffic Light	Traffic Sign	Vegetation	Terrain	Sky	Person	Rider	Car	Truck	Bus	Train	Motorcycle	Bicycle
ESPNet V2 [36]	66.2	97.3	78.6	88.8	43.5	42.1	49.3	52.6	60.0	90.5	66.8	93.3	72.9	53.1	91.8	53.0	65.9	53.2	44.2	59.9
FastSCNN [18]	68.0	97.9	81.6	89.7	46.4	48.6	48.3	53.0	60.0	90.7	67.2	94.3	74.0	54.6	93.0	57.4	65.5	58.2	50.0	61.2
ESNet [13]	69.1	97.1	78.5	90.4	46.5	48.1	60.1	60.4	70.9	91.1	59.9	93.2	74.3	51.8	92.3	61.0	72.3	51.0	43.3	70.2
LRDNet [14]	70.1	97.9	81.7	90.4	43.0	49.1	58.9	63.7	66.8	92.1	69.5	94.6	77.6	58.4	93.3	53.2	62.5	59.3	54.5	65.9
JPANet [25]	71.6	98.1	82.7	90.6	47.1	48.2	53.1	60.2	66.1	91.6	69.2	94.5	78.6	60.5	94.0	64.2	75.9	66.5	**63.7**	65.9
LEANet [17]	71.9	98.1	82.7	91.0	51.0	53.2	58.8	65.9	70.3	92.5	70.5	94.3	81.6	59.9	94.1	52.3	68.2	57.2	55.5	69.8
LAANet [15]	73.6	97.9	82.9	91.0	47.5	51.5	59.3	66.0	70.3	92.3	69.9	94.7	81.8	61.4	94.2	58.6	**79.5**	**75.1**	54.3	69.4
Ours	**75.9**	**98.4**	**84.7**	**92.3**	**53.6**	**57.6**	**60.9**	**69.3**	**73.0**	**93.0**	**70.8**	**95.2**	**83.3**	**65.8**	**95.1**	**66.2**	78.9	71.5	61.0	**72.0**

Bold values indicate that the corresponding indicator is superior to other methods.

**Table 7:** Comparison with other methods on the CamVid test set.

Model	Params (M)	MIoU (%)
ERFNet [16]	2.06	63.7
DFFNet [11]	1.9	64.7
CGNet [41]	**0.5**	65.5
MIFNet [12]	2.0	65.7
SwiftNet [35]	12.9	65.7
EFRNet [24]	2.43	66.2
PCNet [40]	1.6	67.0
JPANet-G [25]	3.49	67.5
LEANet [17]	0.74	67.5
AGLNet [38]	1.12	69.4
Ours	1.27	**69.9**

Bold values indicate that the corresponding indicator is superior to other methods.

## Data Availability

The data presented in this study are available on request from the corresponding author.
